# Perimenopause Amelioration of a TCM Recipe Composed of Radix Astragali, Radix Angelicae Sinensis, and Folium Epimedii: An *In Vivo* Study on Natural Aging Rat Model

**DOI:** 10.1155/2013/747240

**Published:** 2013-12-16

**Authors:** Ji-Yan Su, Qing-Feng Xie, Wei-Jin Liu, Ping Lai, Dan-Dan Liu, Li-Hai Tang, Tina T. X. Dong, Zi-Ren Su, Karl W. K. Tsim, Xiao-Ping Lai, Kun-Yin Li

**Affiliations:** ^1^School of Chinese Materia Medica, Guangzhou University of Chinese Medicine, Guangzhou, Guangdong 510006, China; ^2^Central Laboratory of the Second Affiliated Hospital, Guangzhou University of Chinese Medicine, Guangzhou, Guangdong 510006, China; ^3^Department of Biology and Center for Chinese Medicine, Hong Kong University of Science and Technology, Clear Water Bay Road, Hong Kong; ^4^Dongguan Mathematical Engineering Academy of Chinese Medicine, Guangzhou University of Chinese Medicine, Dongguan, Guangdong 523808, China; ^5^The First Affiliated Hospital of Chinese Medicine, Guangzhou University of Chinese Medicine, Guangzhou, Guangdong 510405, China

## Abstract

Traditional Chinese medicine (TCM) has been extensively applied as preferable herbal remedy for menopausal symptoms. In the present work, the potential of a TCM recipe named RRF, composed of Radix Astragali, Radix Angelicae Sinensis, and Folium Epimedii, was investigated on a natural aging rat model. After administration of RRF (141, 282, and 564 mg/kg/d), the circulated estradiol (E_2_) level increased accompanied by a reduction of serum follicle stimulating hormone (FSH). But no significant impact on serum lutenizing hormone (LH) level was observed. As a result of the E_2_-FSH-LH adjustment, the histomorphology degenerations of ovary, uterus, and vagina of the 11.5-month female rats were alleviated. And lumbar vertebrae trabecular microstructure was also restored under RRF exposure by means of increasing the trabecular area and area rate. Moreover, levels of hypothalamic dopamine (DA) and norepinephrine (NE) rallied significantly after RRF treatment. Results from our studies suggest that RRF possesses a positive regulation on the estrogen imbalance and neurotransmitter disorder, thereby restoring reproductive organ degeneration and skeleton deterioration. The above-mentioned benefits of RRF on the menopause syndromes recommend RRF as a potential candidate for the treatment of perimenopausal syndrome.

## 1. Introduction

Natural menopause is the permanent cessation of menstruation resulting from the loss of ovarian follicular activity for mid-age women [1]. But the lasting perimenopause stage bothers over 50% of the world women population from the onset to cessation. It not only causes a confusing complexity of nonspecific somatic and psychological symptoms, including hot flashes, sweating, anxiety, and mood swings, but also predisposes a range of medical issues such as coronary heart disease, osteoporotic fractures [2], and sexual health concerns [3]. Since all of these could be attributed to estrogen deficiency and the consequent hormonal dysregulation resulting from the sharp decline of ovarian supply of germ cells and the dramatic endocrine function change [4], physicians have introduced hormone replacement therapy (HRT) as the prevailing therapeutic regime to allay series of perimenopausal symptoms 30 years ago [5]. But HRT is getting opposed by both women patients and their health-care physician due to its risks associated with endometrium proliferation and cancer [6, 7], breast cancer [8], and cardiovascular disease [9]. On the other hand, as a safer alternative for HRT, herbal medicine has been considered as a better complimentary therapy for its evident base with thousands-year history in Asia, such as China, Korea, and Japan [9–11], particularly supported by their attenuation on perimenopausal symptoms [12].

In China, there is a long history of treating gynaecological disorders/conditions with herbal medicine products (HMPs) based on the theory of traditional Chinese medicines (TCMs). Among the famous TCM formulas, one named *Danggui Buxue Tang* (DBT), firstly prescribed by Li Dongyuan in *Neiwaishang Bianhuo Lun* in AD 1247, has been widely used for women in gynaecology, especially those with menopausal symptoms. This formula is designed to raise *Qi* (vital energy) and nourish *Blood* (body circulation) consisting of Radix Astragali (root of *Astragalus membranaceus* (Fisch.) Bge. var. *mongholicus* (Bge.) Hsiao, Leguminosae) and Radix Angelicae Sinensis (root of *Angelica Sinensis* (Oliv.) Diels, Apiaceae). Previous pharmacological results suggested that DBT displays an estrogen-like activity on receptor phosphorylation without proliferating mammary gland cells [13], in addition to multiple abilities to promote hematopoietic function [14, 15], to prevent osteoporosis [16], and to stimulate immune response [17, 18]. Interestingly, our previous study on an ovariectomized model revealed that a herbal recipe (named RRF) derived from DBT, which includes Radix Astragali, Radix Angelicae Sinensis, and Folium Epimedii (leaves of *Epimedium brevicornum* Maxim, Berberidaceae), had a potential efficacy on both estrogen regulation and bone formation promotion [19], in which RRF exhibited the abilities to relieve the dramatic sexual hormonal unbalance of estradiol (E_2_), lutenizing hormone (LH), and follicle stimulating hormone (FSH) and to restore the bone mineral density (BMD). All these findings strongly indicated the potential of RRF on prevention and treatment of women's perimenopausal symptoms.

In the present study, we further explored the pharmacological action of RRF on a natural aging female rat model. Skeleton and reproductive organs assessments were performed to evaluate the degeneration alleviation by RRF. Levels of sexual hormone including estradiol (E_2_), follicle stimulating hormone (FSH), and lutenizing hormone (LH) were quantified to confirm this alleviation. Indices of dopaminergic and serotonergic activity, that is, *β*-endorphin (*β*-EP), norepinephrine (NE), dopamine (DA), 5-hydroxytryptamine (5-HT), and 5-hydroxyindoleacetic acid (5-HIAA), were also determined to investigate RRF's effects on the perimenopause-associated hypothalamus-pituitary-ovarian hormones. The present study aimed to investigate possible regulation of RRF on the dramatic alterations in the setting of estrogen deficiency.

## 2. Materials and Methods

### 2.1. Plant Materials and Preparation of RRF

*Plant Materials*. Radix Astragali (RA), the root of *Astragalus membranaceus *(Fisch.) Bge. var. *mongholicus* (Bge.) Hsiao, Leguminosae, and Radix Angelicae Sinensis (RAS), the root of *Angelica Sinensis* (Oliv.) Diels, Apiaceae, were purchased from Shanxi Hunyuan Wangsheng Astragalus Development Co., Ltd. (Lot. 070519) and Guangxi Xinlong Pharmaceutical Co., Ltd. (Lot. 070615), respectively. Folium Epimedii (FE), the leaves of *Epimedium brevicornum *Maxim, Berberidaceae, was obtained from Longxi county in Gansu province (Lot. 070715). All plant materials were authenticated by Professor Zi-Ren Su. The authenticated voucher specimens (Voucher no. 07-05-25 for RA, Voucher # 07-06-20 for RAS, and Voucher # 07-07-20 for FE) were kept in the School of Chinese Materia Medica, Guangzhou University of Chinese Medicine. Assurance of quality control for all the materials was validated according to the Chinese Pharmacopeia (China Pharmacopoeia Committee, 2010) and certified to be within the permitted range of heavy metal and bacterial contamination, by independent government approved service laboratories.

*Preparation of RRF*. RRF was prepared as described previously [19]. Amounts of RA, RAS, and FE were weighed according to a ratio of 5 : 1 : 5. RAS and RA were ground, mixed by vortex, and then extracted twice, each with 8 times of boiling water (800 mL per 100 g mixture) for 2 h. The water extracts were pooled, concentrated (RD 1.18~1.22, 60°C), and centrifuged for 0.5 h at 15000 rpm at room temperature. The obtained supernatant was reconcentrated (RD 1.35~1.40, 60°C) and dried under vacuum to get RA-RAS extract. The dried and powered aerial part of FE was thoroughly extracted twice, each with 7 times of 70% ethanol (700 mL per 100 g powder) for 1 h. The ethanol extracts were pooled, concentrated (RD 1.10~1.15, 60°C), and centrifuged for 0.5 h at 15000 rpm at room temperature. The resulting supernatant was reconcentrated (RD 1.35~1.40, 60°C) under vacuum and dried out by vacuum drying technique. The two dry extracts were blended thoroughly with an appropriate amount of microcrystalline cellulose and cross-linking polyvinylpyrrolidone (PVP) to produce RRF samples for each experiment. Assurance of quality control has been described previously [20]. HPLC assays as described previously had been employed for assurance of quality control of the typical chemicals of the three herbs, astragaloside IV for RA, ferulic acid for RAS, and icariin for FE. Details were shown in the supporting information of the previous paper [19].

### 2.2. Chemicals and Reagents

Reference drug Premarin (conjugated equine estrogens (CEE)) was bought from Wyeth Pharmaceuticals Inc. (Lot. 071207). Estradiol (E_2_), luteotropin (LH), and follicle stimulating hormone (FSH) kits were purchased from Beijing Kemei biotechnology Co., Ltd. (Lot. 081025). *β*-Endorphin (*β*-EP) kit was from Beijing Puerweiye Biotechnology Co., Ltd. (Lot. 081024). Reference chemical standards of 5-hydroxyindoleacetic acid (5-HIAA), 5-hydroxytryptamine (5-HT), dopamine (DA), and norepinephrine (NE) were kindly provided by Hong Kong University of Science and Technology. Methanol of AR grade was purchased from Merck Co., Ltd. (Lot. 1432407 821). H_3_PO_4_ of GR grade was obtained from Guangdong Guanghua Chemical Works Co., Ltd. (Lot. 20061201), NaOH of GR grade was obtained from Tianjin Baishi Chemical Industry Co. Ltd. (Lot. 20050730), and D(+)-10-camphorsulfonic acid of GR grade was obtained from National Pharmacy Group Chemical Reagent Co., Ltd. (Lot. 20080326). HClO_3_ of GR grade was obtained from National Pharmacy Group Chemical Reagent Co., Ltd. (Lot. 20080702). Chloral hydrate was obtained from Tianjin Kermel Chemical Reagent Co., Ltd. (Lot. 20070823). NaH_2_PO_4_ of GR grade was obtained from Tianjin Damao Chemical Reagent Works (Lot. 20081126). Deionized water was prepared by a Millipore water purification system (GenPure, TKA Co., Ltd.).

### 2.3. Animals

Forty special-pathogen-free (SPF) 11-month female Sprague-Dawley rats (body weight 180 ± 20 g) were obtained from the Medical Experiment Animal Center of Guangzhou University of Chinese Medicine. Licence for rats was SCXK (YUE) 2003-0001. All animals were maintained under environmentally controlled conditions of 23–25°C and 12 h light/12 h dark cycle and received humane care in accordance with the guide for the care and use of laboratory animals, published by the US National Institution of Health (NIH Publication, revised in 1985). All experimental protocols involving animals and their care were approved by our institutional animal research ethics committee with reference to the European community guidelines and the regulations of the National Institute of Health of USA.

### 2.4. Treatments and Sample Preparation

15 days after acclimation, the aged rats (11.5-month) were body-weight-matched and randomly divided into control group (equal volume of normal saline, *p.o.*), CEE group (0.1 mg/kg/d, *p.o.*), and RRF groups (doses of 141, 282, and 564 mg/kg/d, resp., *p.o.*). After 16-week administration (once a day), rats were fasted for 12 hours after the last administration. On the next day, they were weighed and subjected to bone mineral density (BMD) assessment under anesthesia with 4% chloral hydrate. On the third day, they were blooded from aorta abdominalis and then sacrificed by decollation immediately for hypothalamuses isolation (on ice). The second to fourth lumbar vertebra trabeculae were dissected for trabecula architecture assay. Ovary, uterus, and vagina were harvested for histomorphology observation. The dissected hypothalamuses were frozen in liquid nitrogen and stored at −80°C for HPLC-ECD assays.

### 2.5. Assessment of BMD

BMD assessments of the whole body, lumbar vertebrae, both femoral necks, and both femurs were conducted by dual-energy X-ray absorptiometry (DEXA) on Hologic QDR-4500 A X-ray bone densitometer (Lunar Prodigy, GE Co., Ltd.). The scan field size was 5.08 × 1.902 cm, resolution was 0.0254 × 0.0127 cm, and scan speed was 7.25 mm/second. The volumetric BMDs (g/cm^3^) of total body, lumbar vertebrae, femurs, and femoral necks were all analyzed using the built-in software program as measuring densitometry parameters.

### 2.6. Lumbar Vertebra Trabecula Microstructure Assay

The obtained lumbar vertebra trabeculae were fixed by 10% formaldehyde, and the specimens were then embedded in paraffin and stained by hematoxylin-eosin. Trabecular area and trabecular area rate were recorded using the Olympus biomicroscope (BX50) equipped with a computerized semiautomatic image analysis system (Cosmozone II; Nikon, Tokyo, Japan), as described previously [21–25]. Three fields of each section (×10) were selected for trabecular counting.

### 2.7. Histomorphology Assessment of Ovary, Uterus, and Vagina

The harvested ovary, uterus, and vagina were weighed for organ index measurement (organ wet weight (g)/body weight (g)%) and then subjected to 10% formaldehyde fixation, paraffin embedding, and hematoxylin-eosin staining for histomorphology observation.

### 2.8. Radioimmunoassay (RIA) for E_2_, LH, FSH, and *β*-EP

The collected blood samples were divided into two parts. One part was mixed with 15 *μ*L Na_2_EDTA and 20 *μ*L aprotinin and centrifuged at 4°C at 4000 rpm for 10 min, to separate plasma. The other coagulated for 2 hours and was centrifuged at 4°C at 4000 rpm for 10 min to separate serum. The separated plasma for *β*-EP assay and serum for determination of E_2_, FSH, and LH were stored at −80°C immediately until radioimmunoassay according to the kit manuals.

### 2.9. HPLC-ECD Determination for DA, NE, 5-HT, and 5-HIAA

Determination of the neurotransmitters was performed as described previously [19]. The harvested hypothalamuses were homogenized and deproteinized in ice-cold 0.1 M perchloric acid, containing 3,4-dihydroxybenzylamine hydrobromide as an internal standard. The homogenates were centrifuged at 18000 rpm for 20 min at 4°C, and the obtained supernatants were collected, filtered with 0.22 *μ*m millipore filters, and stored at −80°C for reverse-phase ion pair HPLC-ECD determination. Chromatographic separations were performed on 15 cm Phenomenex (Torrance, CA) column packed with 5 *μ*m particles. The mobile phase solution consisted of 1 mM D(+)-10-camphorsulfonic acid, 100 mM NaH_2_PO_4_, 1 mM Na_2_EDTA, and 5% methanol, adjusted to pH 4.1 with saturated citric acid. The mobile phase was filtered through a 0.22 *μ*m filter, degassed, and delivered at a flow rate of 1.0 mL/min. The inject volume was 10 *μ*L. The identification and purity of the chromatographic peaks, as well as their quantitative evaluation, were performed with respect to peaks obtained from external standards (DA, NE, 5-HT, and 5-HIAA).

### 2.10. Statistical Analysis

Data were presented as means ± standard error of mean (SEM) for the indicated number of independently performed experiments using the SPSS package (SPSS 17.0 for Windows). The statistical significances within parameters were evaluated by one-way analysis of variation (ANOVA). The significant differences (as shown in the plots) were classified as * for *P* < 0.05 and more significant ** for *P* < 0.01.

## 3. Results

### 3.1. RRF Prevented Degeneration of Ovary, Uterus, and Vagina

**Figure 1:**
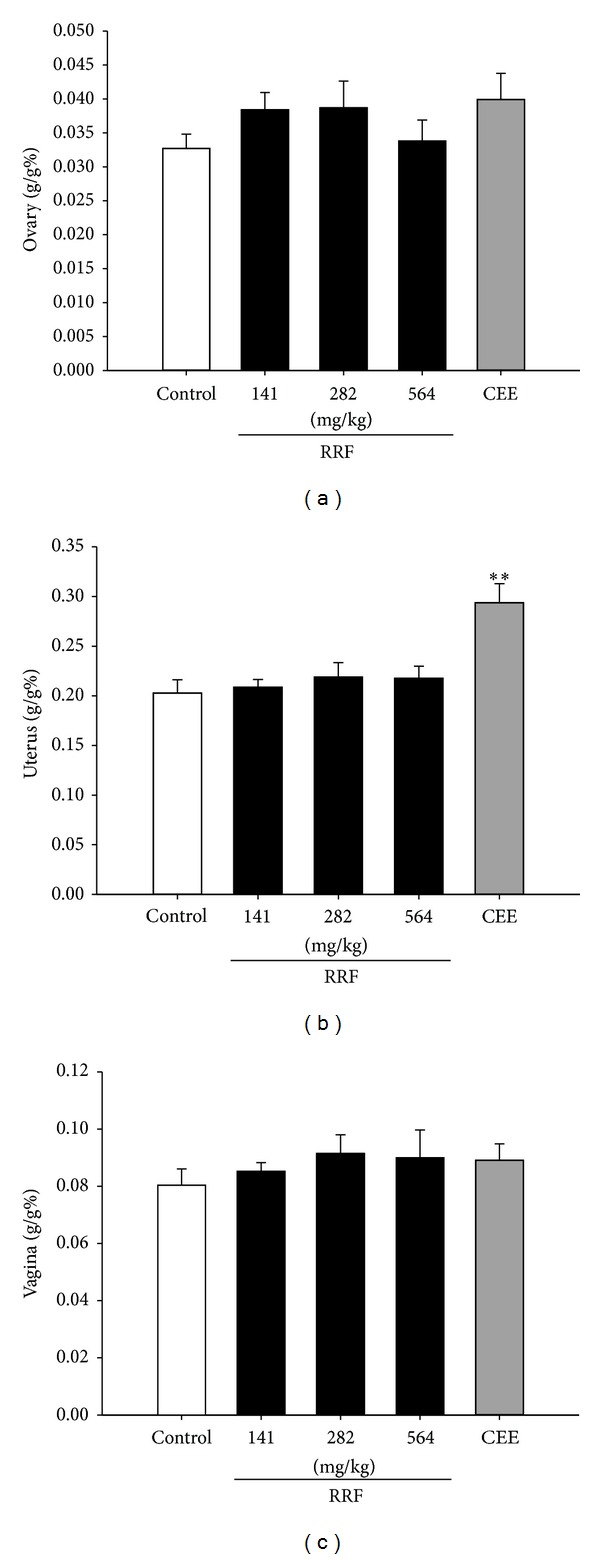
Organ indexes of ovary (a), uterus (b), and vagina (c) in control group (blank bars), CEE group (0.1 mg/kg/d, dark-grey bars), and RRF groups (141, 282, and 564 mg/kg/d, solid-dark bars). Vertical bars represent standard errors of the means, where *n* = 8. Asterisks designate significant differences: *P < 0.05 versus control group and **P < 0.01 versus control group.

**Figure 2:**
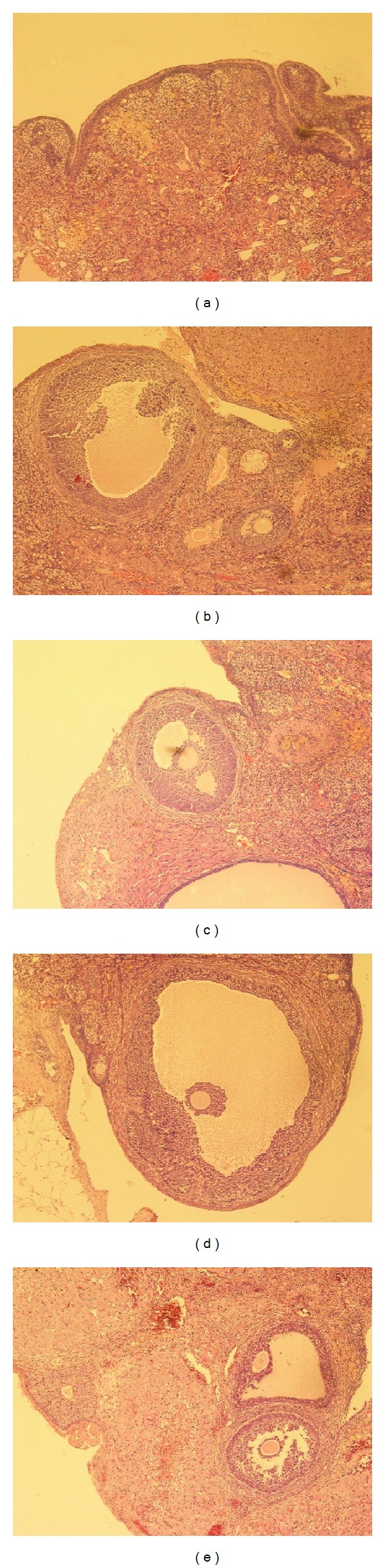
Typical photomicrographs (×100) of the ovary of natural aging rats in control group (a), CEE group (b), and RRF groups ((c), (d), and (e) for 141, 282, and 564 mg/kg/d, resp.).

Although no evident organ indices difference was observed after CEE or RRF treatment (Figure 1), RRF could apparently restore the degeneration of the reproductive organs. Ovary from the untreated aged rats presented the typical senescent condition with few follicles (primary follicles, secondary follicles, or follicles in other stages) and few corpus luteum (Figure 2(a)). By contrast, administration of CEE and RRF significantly increased follicles in primordial, primary, and secondary stages, as well as the corpus luteum present in most parts of the ovary, indicating a beneficial effect on this reproductive organ (Figures 2(b), 2(c), 2(d), and 2(e)).

**Figure 3:**
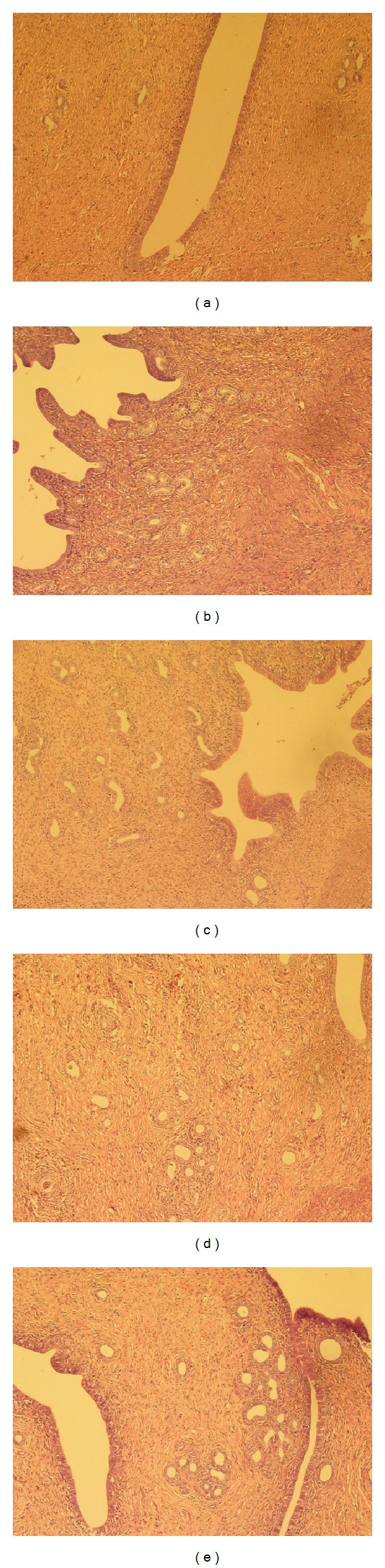
Typical photomicrographs (×100) of the uterus of natural aging rats in control group (a), CEE group (b), and RRF groups ((c), (d), and (e) for 141, 282, and 564 mg/kg/d, resp.).

In terms of uterus, the untreated natural aged rats revealed a typical degenerative condition with thin serosa, muscularis, and compact stroma (Figure 3(a)). The atrophied glands were distributed sparsely in the uneven-depth endometrium and the vascularity was poor. Administration of CEE stimulated all the uterine structures (Figure 3(b)) as indicated by the increase of endometrium thickness and large-cavity glands in proliferative or secretory stages. And structure of uterus wall was clear with replete smooth muscle. Similarly, RRF treatment stimulated the histoarchitecture of the uterus (Figures 3(c), 3(d), and 3(e)) as well, in which there existed more well-developed myometrium smooth muscle cells with red-stained cytoplasm. Proliferative or secretory glands in the endometrium were distributed much more evenly.

**Figure 4:**
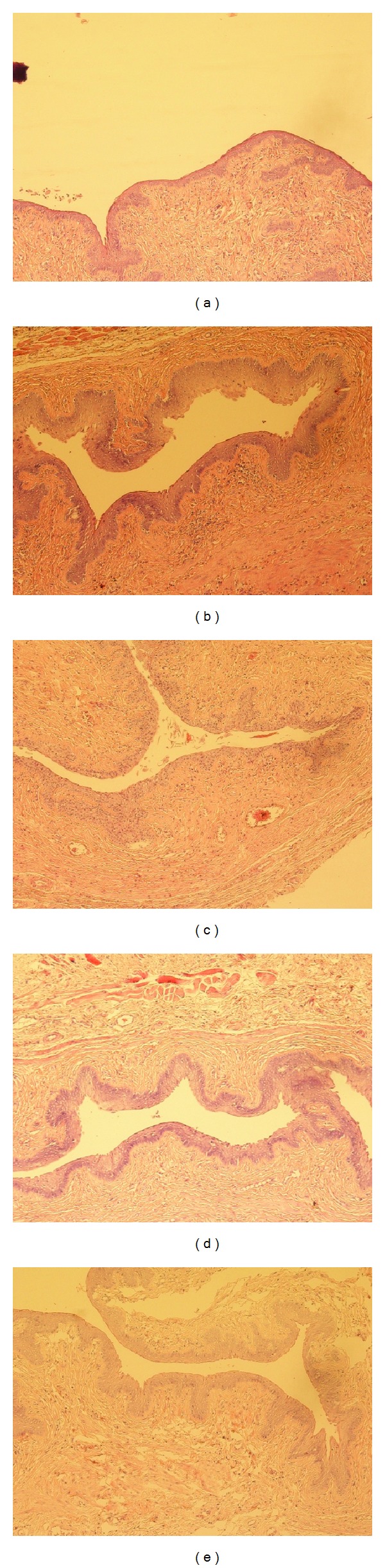
Typical photomicrographs (×100) of the vagina of natural aging rats in control group (a), CEE group (b), and RRF groups ((c), (d), and (e) for 141, 282, and 564 mg/kg/d, resp.).

With regard to vagina, control group displayed the typical atrophic features in mucous with thinner stratified applanate epithelium, basophilic superficial cells, and apparent keratinization (Figure 4(a)). CEE group had shown evident duplicature accompanied with mild keratinization, and the stratified applanate epithelium cells were structure clear and of normal amount and size (Figure 4(b)). Alike promotion could also be observed in RRF groups. The stratified applanate epithelium layers were thicker than those of control group, with more well-developed cells and more evident keratinization (Figures 4(c), 4(d), and 4(e)).

### 3.2. RRF Improved Lumbar Vertebrae Degeneration

Despite the fact that neither CEE nor RRF had obvious effect on BMDs of the aged rats (Table 1), histoarchitecture assay suggests a protection by RRF on lumbar vertebra trabeculae. In comparison to the untreated counterpart, although RRF-administrated rats did not show evident increasing trabecular number (Figure 7(a)), they had substantial improvement in trabecular area (Figure 7(b)) and trabecular area rate (Figure 7(c)). This indicated the reversion potential of RRF on bone degradation, especially the lumbar vertebrae degeneration exacerbating as aging.

### 3.3. RRF Regulated Circulating E_2_, LH, and FSH

**Figure 5:**
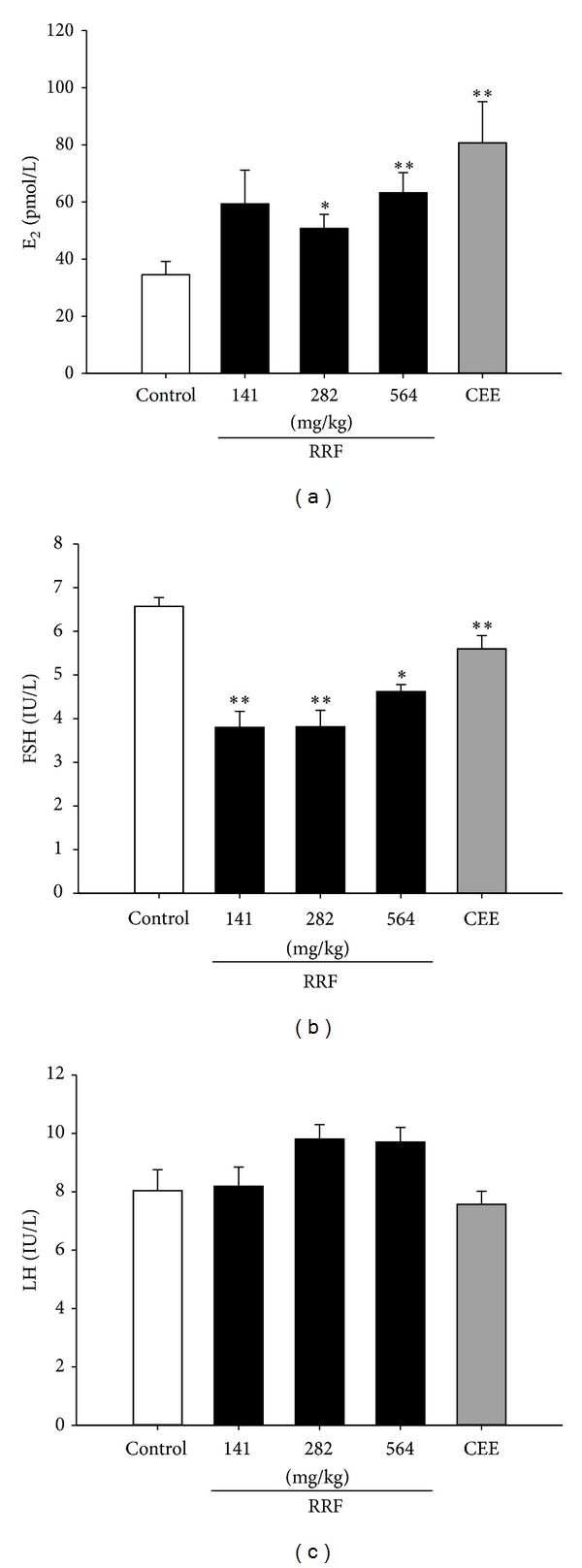
Serum levels of E_2_ (a), FSH (b), and LH (c) in control group (blank bars), CEE group (0.1 mg/kg/d, dark-grey bars), and RRF groups (141, 282, and 564 mg/kg/d, solid-dark bars). Vertical bars represent standard errors of the means, where *n* = 8. Asterisks designate significant differences: *P < 0.05 versus control group and **P < 0.01 versus control group.

As depicted in many literatures, the circulating E_2_ was at a low level accompanied with a high content of circulating FSH in the natural aged rats. By contrast, CEE treatment remarkably elevated the circulating E_2_ level (Figure 5(a)), while it significantly decreased that of FSH (Figure 5(b)). Similarly, RRF exposure substantially promoted the content of E_2_ (Figure 5(a)) but dramatically depressed FSH (Figure 5(b)). However, neither CEE nor RRF affected the LH level of the subject aged SD rats. These result shows that RRF positively regulated the imbalanced female hormone in natural aged rat.

### 3.4. RRF Modulated Neurotransmitters

**Figure 6:**
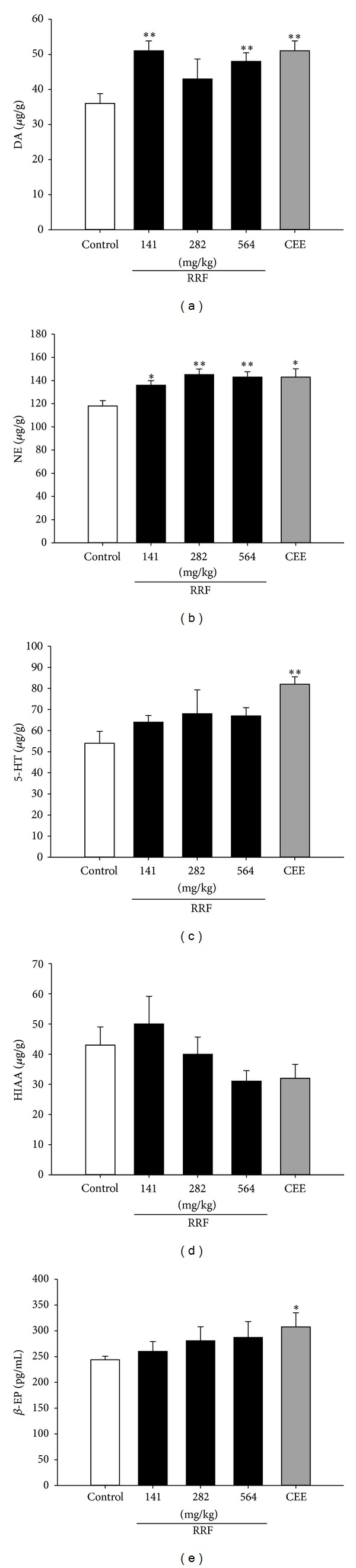
Hypothalamus levels of DA (a), NE (b), 5-HT (c), 5-HIAA (d), and *β*-EP (e) in control group (blank bars), CEE group (0.1 mg/kg/d, dark-grey bars), and RRF groups (141, 282, and 564 mg/kg/d, solid-dark bars). Vertical bars represent standard errors of the means, where *n* = 8. Asterisks designate significant differences: *P < 0.05 versus control group and **P < 0.01 versus control group.

**Figure 7:**
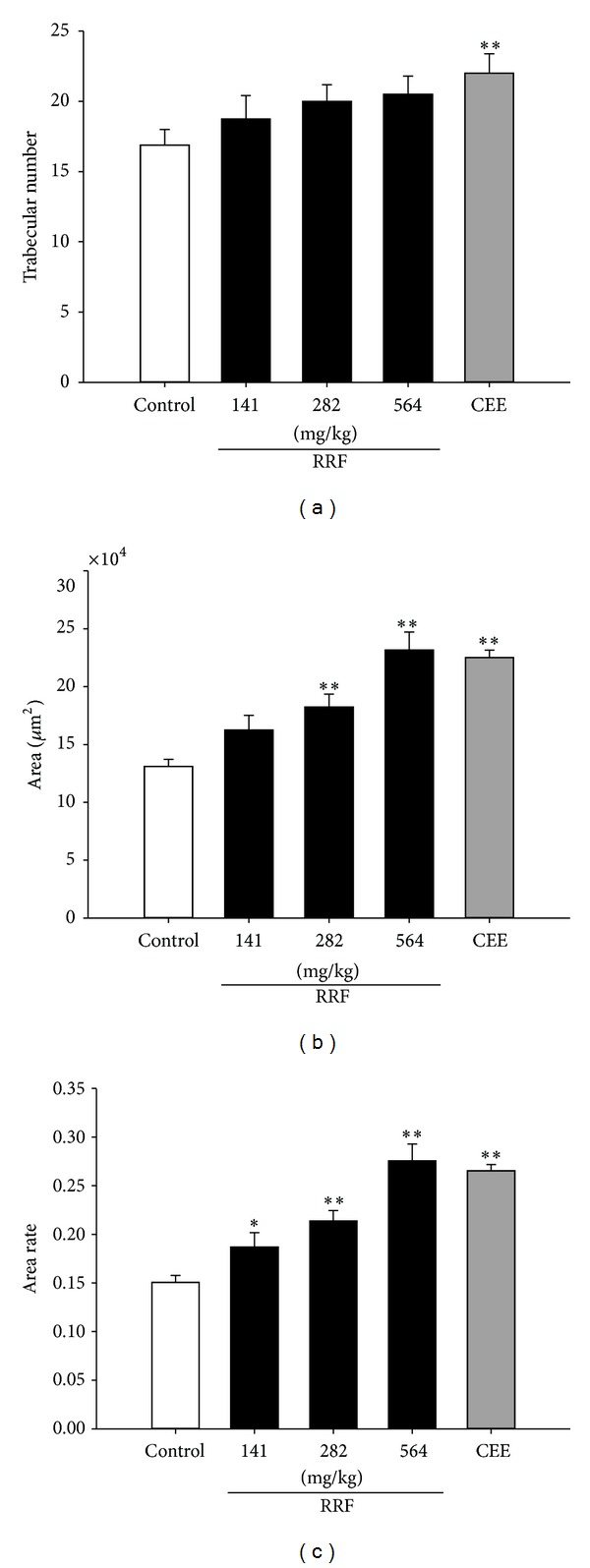
Histoarchitecture of lumbar vertebra trabeculae. The trabecular number (a), area (b), and area ratio (c) in control group (blank bars), CEE group (0.1 mg/kg/d, dark-grey bars), and RRF groups (141, 282, and 564 mg/kg/d, solid-dark bars). Vertical bars represent standard errors of the means, where *n* = 8. Asterisks designate significant differences: *P < 0.05 versus control group and **P < 0.01 versus control group.

As depicted in Figure 6, the aged SD rats showed lower levels of DA, NE, 5-HT, and *β*-EP, while their counterparts administrated with CEE had significantly higher level of the above-mentioned neurotransmitters. RRF treatment had similar effect on the catecholaminergic neurotransmitters. In detail, it evidently upregulated DA (Figure 6(a)) and its derivative NE (Figure 6(b)). However, RRF had no impact on 5-HT, 5-HIAA, or *β*-EP (Figures 6(c), 6(d), and 6(e)). The aforementioned data suggested that RRF would have favorable effect on the nervous system during perimenopause.

## 4. Discussion

During perimenopause, reproduction organ degeneration and skeleton deterioration are two main manifestations of perimenopause syndrome [1]. As shown in our work, RRF exhibited apparent restoration on the aged rats' reproduction organs, as being manifested by (1) restored amounts of follicle and corpora lutea in ovary, (2) thickened uterine wall and increasing endometrium gland present in proliferative or secretory stages, and (3) reversed mucosa atrophy in vagina and recurring well-developed stratified applanate epithelium. Additionally, although no significant BMD regain was observed, RRF did bring benefit to the lumbar vertebrae trabecular architecture by increasing the trabecular area and area rate, indicating that RRF could inhibit the overwhelming osteoclast reabsorption and facilitate the osteoblast formation. All these suggest a potential degeneration amelioration of RRF to the menopause-suffering women.

When stepping into the later phase of menopause, women experience a stage when follicles diminish sharply due to the accelerated depletion of oocytes and become less responsive to FSH stimulation over the aging process [1]. The above opinions imply that reproduction organ degeneration and skeleton deterioration are closely related to female hormone imbalance, particularly the estrogen deficiency. The dramatic descension of E_2_ and elevation in FSH and LH are characteristics of a dampening of the positive hypothalamic-pituitary feedback response to estrogen [26, 27], finally causing a panel of perimenopause syndrome [4] concomitantly with the declining ovarian reserve [28–30]. One more serious outcome of estrogen deficiency is its disruption on the bone remodeling process, via an initial wave of T cell activation and the surge of cytokines, that is, tumor necrosis factor-*α*, interferon-*γ*, and interleukin-1, not only by accelerating osteoclasts formation, but also by the direct repressive effects on osteoblasts [31]. Therefore, it is necessary to investigate whether the degeneration prevention of RRF is concurrent with a regulation on the female hormone. In the present study, RRF did exert a significant increase in the serum level of E_2_ along with an extremely remarkable decrease of FSH, indicating that, via the E_2_-FSH-LH adjustment, RRF would relieve the organ and skeleton deterioration, thereby ameliorating the perimenopause syndrome.

Another irrefragable fact in this field is that the change of ovarian activity can influence hypothalamic and pituitary function through the hypothalamic-pituitary-sexual gland axis [32, 33]. This is mainly due to the estrogen receptors that are widely present in the rat cortex, pituitary, and hypothalamus [34, 35]. Therefore, changes in neurotransmitters are important consequences of the deregulation of gonadal hormone production when many central nervous system activities deteriorate, especially those associated with hippocampal functions [35, 36]. DA and its derivation NE are important catecholaminergic monoamines that are always involved in emotional disorders if their release is inhibited or their pertinent neurons are destroyed [37–39]. Serotonergic neurotransmitters 5-HT and its metabolite 5-HIAA, which are involved in the regulation of such diverse functions as reproduction, mood, sleep, and cognition, have been proved to be altered in part by ovarian hormones [40, 41]. Besides, decrease in plasma *β*-EP levels, the most important endogenous opioid peptide that exerts behavioral, analgesic, thermoregulatory, and neuroendocrine properties, was found to induce hot flushes and sweat episodes in postmenopausal women after surgical or spontaneous menopause [42]. Thus impacts of RRF on these neurotransmitters were investigated for the therapeutic profile understanding. The result showed that, in parallel with the elevated plasma E_2_, RRF significantly increased the catecholaminergic neurotransmitters (DA and NE) levels in a favorable fashion, which were in accordance with the reported neuroprotection and regulation of E_2_ [41, 43–45]. This finding suggests that neurotransmitter regulation is another vital contribution for the perimenopause amelioration of RRF.

Experimental endeavors have proved that DBT, the formula that RRF is derived from, had a meaningful differentiation stimuli on MG-63 [46] and an estrogen-like regulation on MCF-7 but without proliferation on MCF-7 [46, 47]. The point that needs to be noticed from these findings is that they all appreciated the water extract of DBT according to the ancient procedure at the weight ratio of 5 : 1 (RA : RAS), which was proved to consist of polysaccharides [46], as the responsible part for its activity. With regard to FE, it is the flavonoids from FE, which could be accumulated to the most extent by alcohol, that have been reported to possess estrogen-like activity through the neuroendocrine and immune system [48, 49]. As described in Section 2.1, RRF is composed of the water extract of RA and RAS (5 : 1) and the ethanol extract of FE, which are similar to the reported preparations of DBT and FE [46, 48, 49]. Together with the above-mentioned literature, our data indicated that the active principals responsible for the estrogen-like activity of RRF may rely on its polysaccharides and flavonoids. By providing subjects with these estrogen-like compounds, RRF would recover the ovary function of the aged subjects and regulate the E_2_ imbalance, thus alleviating the perimenopause symptoms. Whereas, for the sake of safe application, the exact principals have to be figured out by multitechnologies, including HPLC, gas chromatography and mass spectrometry (GC-MS), nuclear magnetic resonance spectroscopy (NMR), and mechanistically based biochemical and molecular assays.

## 5. Conclusions

Taken together, the outlined results provided reasonable support for the potential benefit of RRF on the menopause syndromes. By the assumed estrogen-like activity, RRF would make a direct upregulation of E_2_ and a following descending of the feedback-released FSH, thereby enhancing the sexual hormone regulation. Consequently, the reproductive organ degeneration and skeleton deterioration were significantly alleviated due to the E_2_-FSH-LH adjustment by RRF. Moreover, dopaminergic neurotransmitter production, DA and NE, was normalized as a result of the positive adjustment on estrogen. Thus, owing to the benefit of sexual hormonal and neurotransmitter regulation, RRF might constitute a novel therapeutic agent for the prevention and treatment of perimenopausal disorders in midlife women, providing a safe and effective complementary or alternative to conventional medication.

## Figures and Tables

**Table 1:** BMD of different parts of the subjected rats after treatment (mean ± SEM, *n* = 8).

Group	Whole body	Lumbar vertebrae	Left femoral neck	Right femoral neck	Left femur	Right femur

Control	0.177 ± 0.003	0.202 ± 0.007	0.228 ± 0.004	0.231 ± 0.005	0.210 ± 0.004	0.209 ± 0.004
RRF (141 mg/kg/d)	0.180 ± 0.002	0.219 ± 0.005	0.218 ± 0.008	0.215 ± 0.007	0.203 ± 0.003	0.207 ± 0.002
RRF (282 mg/kg/d)	0.182 ± 0.010	0.210 ± 0.005	0.220 ± 0.006	0.222 ± 0.007	0.206 ± 0.005	0.216 ± 0.007
RRF (564 mg/kg/d)	0.179 ± 0.002	0.206 ± 0.006	0.226 ± 0.003	0.222 ± 0.003	0.208 ± 0.005	0.209 ± 0.004
CEE (0.1 mg/kg/d)	0.181 ± 0.003	0.204 ± 0.004	0.235 ± 0.005	0.225 ± 0.004	0.211 ± 0.003	0.208 ± 0.003

## Abbreviations

RRF: A derived herbal recipe
RA: Radix Astragali
RAS: Radix Angelicae Sinensis
FE: Folium Epimedii
BMD: Bone mineral density
CEE: Conjugated equine estrogens premarin
FSH: Follicle stimulating hormone
E_2_: Estradiol
LH: Lutenizing hormone
5-HIAA: 5-Hydroxyindoleacetic acid
5-HT: 5-Hydroxytryptamine
DA: Dopamine
NE: Norepinephrine
*β*-EP: *β*-Endorphin
HRT: Hormone replacement therapy.
